# Possible roles of CAHS proteins from Tardigrade in osmotic stress tolerance in mammalian cells

**DOI:** 10.1247/csf.24035

**Published:** 2024-11-19

**Authors:** Takahiro Bino, Yuhei Goto, Gembu Maryu, Kazuharu Arakawa, Kazuhiro Aoki

**Affiliations:** 1 Division of Quantitative Biology, National Institute for Basic Biology, National Institutes of Natural Sciences, 5-1 Higashiyama, Myodaiji-cho, Okazaki, Aichi 444-8787, Japan; 2 Quantitative Biology Research Group, Exploratory Research Center on Life and Living Systems (ExCELLS), National Institutes of Natural Sciences, 5-1 Higashiyama, Myodaiji-cho, Okazaki, Aichi 444-8787, Japan; 3 Department of Basic Biology, School of Life Science, SOKENDAI (The Graduate University for Advanced Studies), 5-1 Higashiyama, Myodaiji-cho, Okazaki, Aichi 444-8787, Japan; 4 Laboratory of Cell Cycle Regulation, Graduate School of Biostudies, Kyoto University, Yoshidakonoe-cho, Sakyo-ku, Kyoto, Kyoto 606-8501, Japan; 5 Institute for Advanced Biosciences, Keio University, Nihonkoku 403-1, Daihoji, Tsuruoka, Yamagata, 997-0017, Japan; 6 Graduate School of Media and Governance, Keio University, Endo 5322, Fujisawa, Kanagawa 252-0882, Japan; 7 Faculty of Environment and Information Studies, Keio University, Endo 5322, Fujisawa, Kanagawa 252-0882, Japan; 8 Center for Living Systems Information Science, Graduate School of Biostudies, Kyoto University, Yoshidakonoe-cho, Sakyo-ku, Kyoto, Kyoto 606-8501, Japan

**Keywords:** anhydrobiosis, Tardigrades, live imaging, disordered proteins, desiccation tolerance

## Abstract

Anhydrobiosis, a phenomenon in which organisms survive extreme dehydration by entering a reversible ametabolic state, is a remarkable example of survival strategies. This study focuses on anhydrobiosis in tardigrades, which are known for their resilience to severe environmental conditions. Tardigrades utilize several protective mechanisms against desiccation, notably the constitutive expression of cytoplasmic abundant heat soluble (CAHS) proteins in *Ramazzottius varieornatus*. These proteins share similarities in their amphiphatic alpha helices with late embryogenesis abundant (LEA) proteins, but differ significantly in their amino acid sequences. In this study, we further explored the functionality of CAHS proteins by analyzing their role in aggregation and tolerance to hyperosmotic stress in mammalian cells. Using live cell imaging, we examined the subcellular localization of several CAHS and LEA proteins in response to hyperosmotic stress. The expression of CAHS1, CAHS3, and CAHS8 tended to enhance the resilience to the hyperosmotic conditions. These findings not only deepen our understanding of the molecular mechanisms of anhydrobiosis but also highlight the potential of CAHS proteins as cryoprotectants.

## Introduction

Anhydrobiosis, derived from “life without water” in Greek, is a phenomenon where an organism loses almost all of its water and enters a state of reversible ametabolism (Crowe *et al.*, 1998; Keilin, 1959). An organism capable of anhydrobiosis tolerates extreme dehydration (desiccation), dries to equilibrium with moderately to extremely dry air, and then restores its vital functions after rehydration. Anhydrobiosis is observed in both microorganisms and animals, including bacteria, nematodes, seeds, spores, gemma, lichen soredium, egg, shoot fragments, and more (García, 2011; Hibshman *et al.*, 2020; Leprince and Buitink, 2015). Desiccation primarily damages proteins, nucleic acids, and membrane lipids, leading to cell death. Therefore, living organisms that exhibit anhydrobiosis have protective mechanisms to mitigate desiccation. Trehalose accumulation (Crowe *et al.*, 1998; Lapinski and Tunnacliffe, 2003), late embryogenesis abundant (LEA) proteins (Goyal *et al.*, 2005; Kikawada *et al.*, 2006; MacRae, 2016), heat shock proteins (Cornette *et al.*, 2010; King and MacRae, 2015), and ROS scavenging (Gusev *et al.*, 2010; Rizzo *et al.*, 2010) have been proposed as mechanisms of desiccation tolerance (Janis *et al.*, 2018).

Tardigrades, widely used as model organisms for anhydrobiosis research, exhibit extreme tolerance to high and low temperatures, high pressure, and radiation in their anhydrobiotic state (Rebecchi *et al.*, 2007; Wright, 2001). Although several reports have been reported on the defense mechanisms underlying anhydrobiosis in tardigrades, including induction of heat shock proteins and defensive peroxidases (Reuner *et al.*, 2010; Rizzo *et al.*, 2010; Schokraie *et al.*, 2011; Yoshida *et al.*, 2022), cytoplasmic abundant heat soluble (CAHS) proteins, identified as a family of highly hydrophilic and heat-soluble proteins, have attracted much attention. CAHS proteins are constitutively expressed in *Ramazzottius varieornatus*, which exhibits strong desiccation tolerance without *de novo* gene expression. On the other hand, in another tardigrade, *Hypsibius exemplaris*, the expression of CAHS proteins is strongly induced under desiccated condition, and these expressions are essential for desiccation tolerance (Arakawa, 2022; Kondo *et al.*, 2015, 2019; Yoshida *et al.*, 2017). CAHS proteins share some similarities with LEA proteins in terms of bioinformatic predictions of a strong tendency for amphiphatic α-helix formation, despite their low amino acid sequence similarity to LEA proteins. Recently, several groups, including ours, have reported that the CAHS proteins demonstrate gelation, droplet, or fibrous structures *in vitro* and in cells (Malki *et al.*, 2022; Tanaka *et al.*, 2022; Veling *et al.*, 2022; Yagi-Utsumi *et al.*, 2021). It has also been shown that the heterologous expression of CAHS proteins suppresses hyperosmotic pressure-induced cell death of budding yeast, bacteria, and *Drosphila* S2 cells (Boothby *et al.*, 2017; Tanaka *et al.*, 2022). However, it remains unclear whether the heterologous expression of CAHS proteins confers hyperosmotic stress tolerance to cultured mammalian cells.

In this study, we show the changes in the subcellular localization of *R. varieornatus* CAHS1, CAHS3, CAHS8, CAHS12, late embryogenesis abundant protein mitochondrial (LEAM), and mitochondrial abundant heat soluble (MAHS) proteins upon hyperosmotic stress stimulation by live cell imaging in mammalian cultured cells. We also investigated the tolerance to hyperosmotic stress by the expression of CAHS proteins, and found that the expression of CAHS1, CAHS3, or CAHS8 rendered the cells tolerant to the hyperosmotic stress.

## Methods

### Plasmid construction

**Fig. 1:**
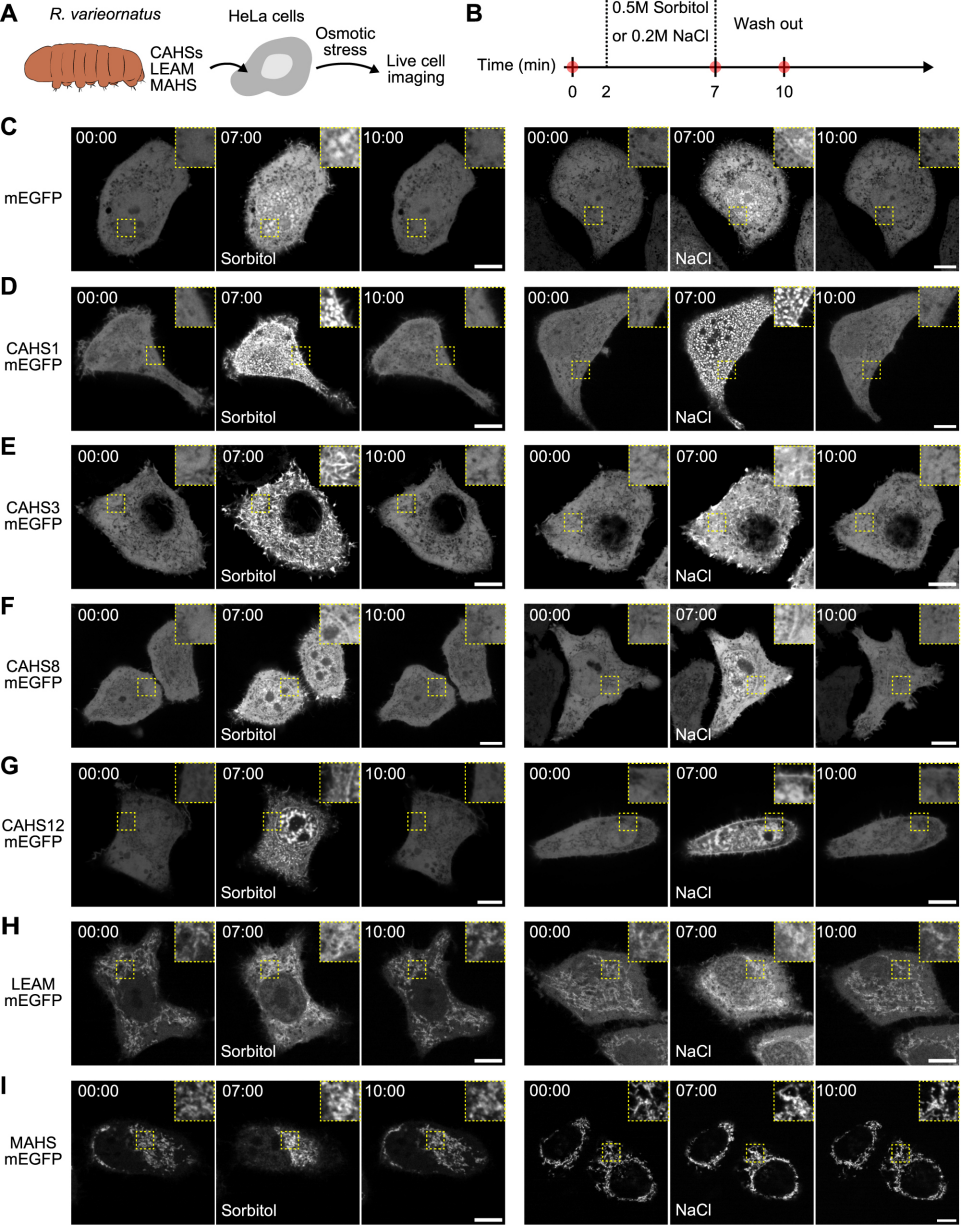
Subcellular localization of CAHS1, CAHS3, CAHS8, CAHS12, LEAM, and MAHS proteins in response to hyperosmotic stress in mammalian cells (A) Schematic representation of the experiments. (B) Timeline of hyperosmotic stress time-lapse imaging. The red dots represent the time points when the representative cells are shown in panels C–I. (C–I) Representative images of HeLa cells overexpressing mEGFP (C), CAHS1-mEGFP (D), CAHS3-mEGFP (E), CAHS8-mEGFP (F), CAHS12-mEGFP (G), LEAM-mEGFP (H), and MAHS-mEGFP (I) proteins at the indicated time points. A solution of 0.5 M sorbitol (left panels) or 0.2 M sodium chloride (right panels) was added after 2 min. Insets are enlarged images of the enclosed area. After 5 min, the cells were washed with fresh medium using a microfluidic system. Scale bar, 10 μm.

**Fig. 2:**
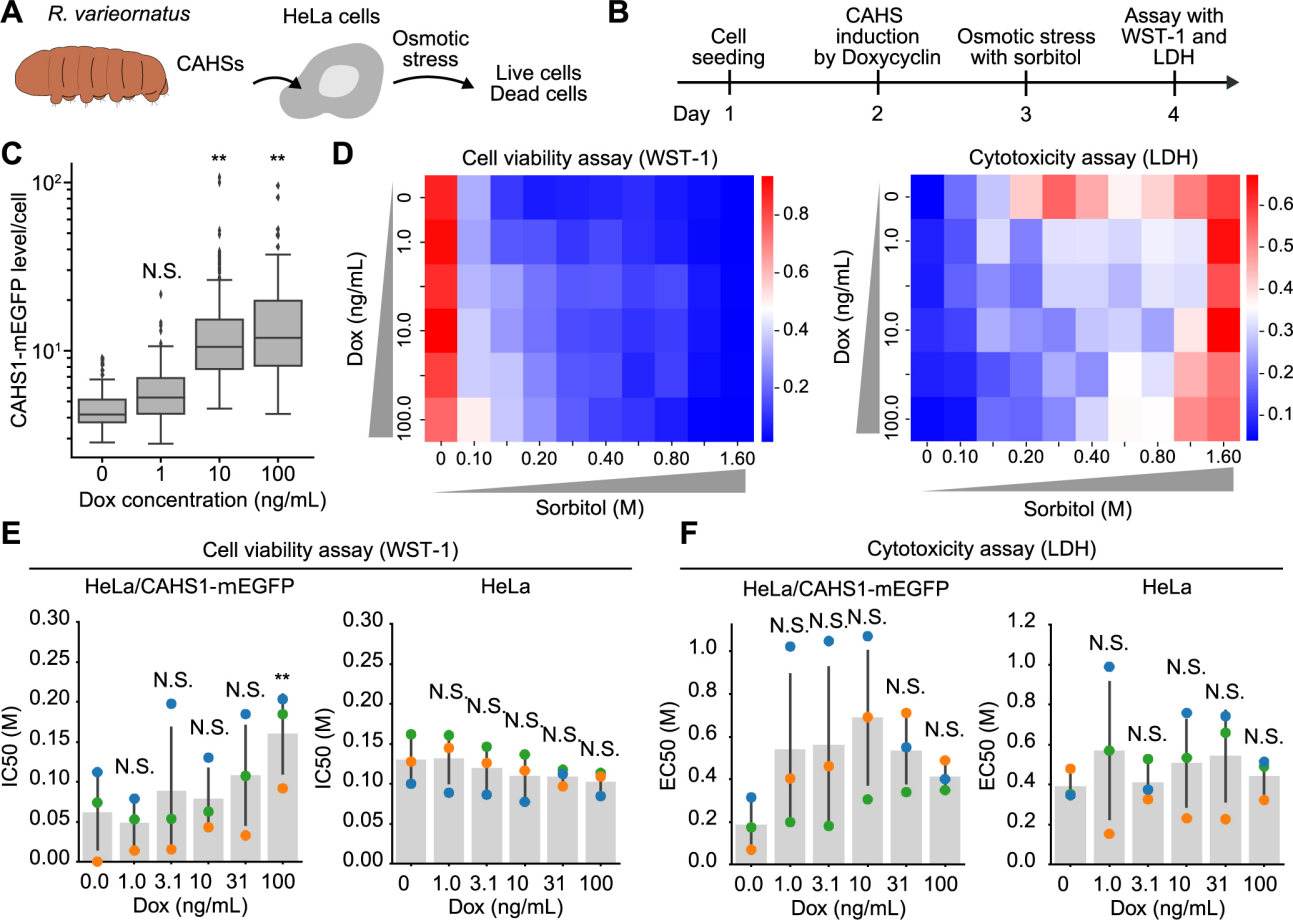
Effects of CAHS1-mEGFP expression on cell viability and cell death upon osmotic stress in HeLa cells (A and B) Schematic representation of the experimental scheme (A) and time course (B) of the stress assays. (C) Doxycycline-induced expression of CAHS1-mEGFP in HeLa cells. HeLa cells were treated with the indicated concentration of doxycycline for 24 h. The cells were imaged by wide-field epi-fluorescence microscopy. The CAHS1-mEGFP expression in each cell is shown as a box plot, in which the box extends from the first to the third quartile with the whiskers denoting 1.5 times the interquartile range. The number of cells is as follows: n = 105 for 0 ng/mL; n = 142 for 1 ng/mL; n = 299 for 10 ng/mL; n = 252 for 100 ng/mL. The symbols indicate the results of Dunnett’s test analysis compared with the 0 ng/mL. **: *p*<0.01, and N.S.: not significant. (D) Heatmap of cell viability assay (left, WST-1 assay) and cytotoxicity assay (right, LDH assay) in HeLa cells expressing CAHS1-mEGFP in a doxycycline-dependent manner. In these heatmaps, red color indicates a higher number of viable cells (WST-1 assay) or dead cells (LDH assay). The average values are shown based on three independent experiments. (E and F) The bar graphs show the mean IC50 values for cell viability (E) and EC50 values for cell death (F) in HeLa cells expressing CAHS1-mEGFP (left) or parental HeLa cells (right) treated with the indicated concentration of doxycycline with S.D. Each dot represents the raw data from three independent experiments. The color (blue, green, and orange) of these dots indicates the data obtained in the same experiments. The symbols indicate the results of *t*-test analysis compared with the 0 ng/mL. **p<0.01, and N.S.: not significant.

**Fig. 3:**
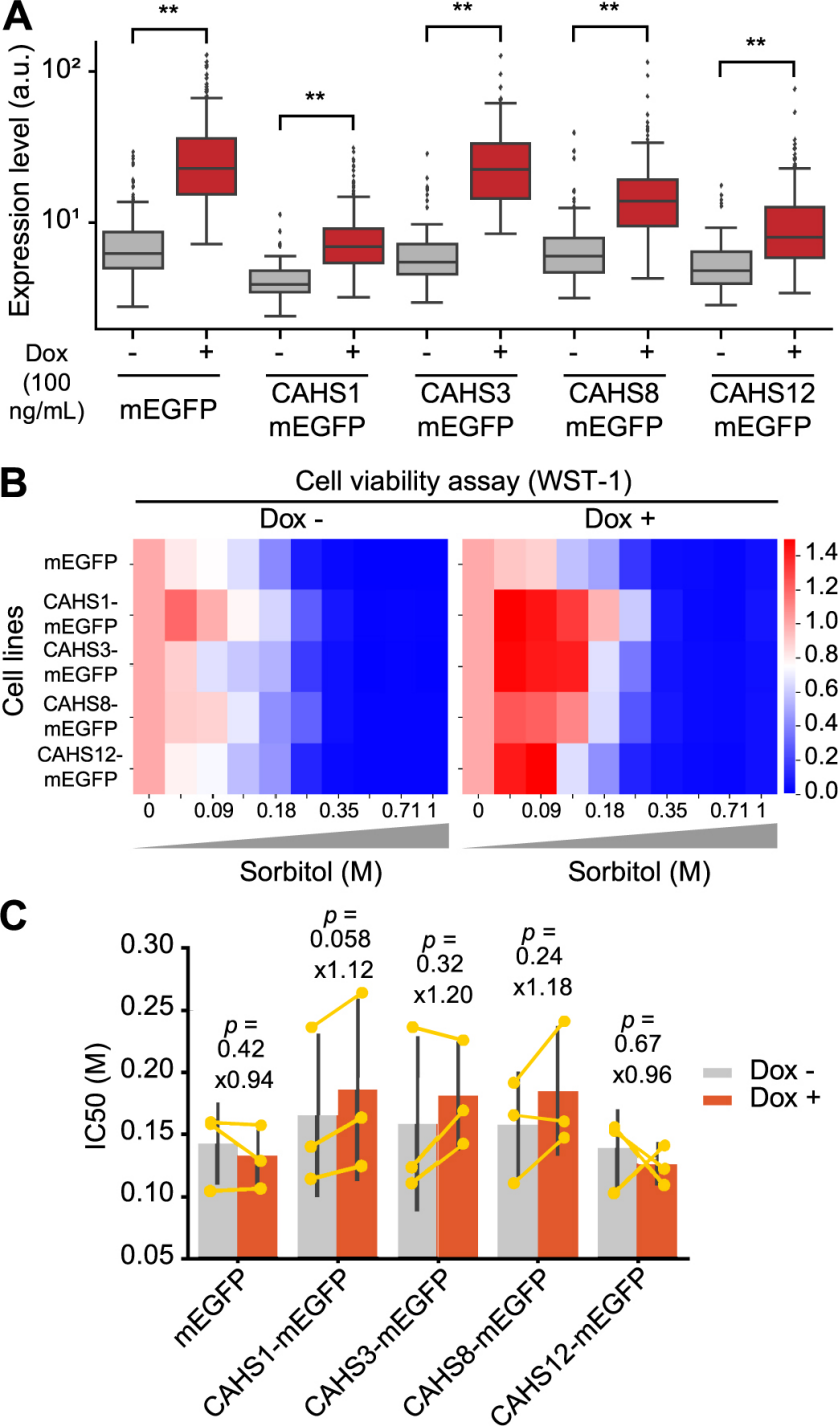
Effects of CAHS3, CAHS8, or CAHS12 expression on cell viability upon osmotic stress in HeLa cells (A) Doxycycline-induced expression of mEGFP, CAHS1-mEGFP, CAHS3-mEGFP, CAHS8-mEGFP, or CAHS12-mEGFP in HeLa cells. HeLa cells were treated with or without 100 ng/mL doxycycline for 24 h. The cells were imaged by wide-field epi-fluorescence microscopy. The mEGFP expression in each cell is shown as a box plot, in which the box extends from the first to the third quartile with the whiskers denoting 1.5 times the interquartile range. The numbers of cells are as follows: mEGFP, n = 238 and 373 (Dox– and Dox+); CAHS1-mEGFP, n = 68 and 298 (Dox– and Dox+); CAHS3-mEGFP, n = 107 and 172 (Dox– and Dox+); CAHS8-mEGFP, n = 245 and 364 (Dox– and Dox+); CAHS12-mEGFP, n = 61 and 286 (Dox– and Dox+). The symbols indicate the results of *t*-test analysis; **p<0.01 compared with the Dox– data. (B) Heatmap of cell viability assay in HeLa cells expressing the indicated proteins without (left) or with 100 ng/mL doxycycline (right). In these heatmaps, red color indicates a higher number of viable cells. The average values are shown based on three independent experiments. (C) The mean of an IC50 value of cell viability against sorbitol before and after doxycycline treatment are shown as a bar graph with S.D. (n = 3). Although *t*-test was conducted for the data compared with the mEGFP data, the statistical significance was not observed.

All plasmids used in this study are summarized in Table S1, along with Benchling links to the plasmid sequences and maps. Plasmids were constructed by standard molecular biology methods. The cDNAs of *R. varieornatus* CAHS1, CAHS3, CAHS8, CAHS12, LEAM, and MAHS were synthesized with codon optimization for the human genome using gBlocks Gene Fragments (Integrated DNA Technologies, Coralville, IA, USA) and inserted into the pCAGGS vector (Niwa *et al.*, 1991) for transient expression experiments (Fig. 1) and the pT2A vector (pT2Apuro-rtTA2-TRE) for drug-inducible expression experiments (Fig. 2 and 3). Of note, since CAHS proteins are known to be abundantly expressed in tardigrades, we optimized the codon of the CAHS genes in order to express sufficient amounts of CAHS proteins in HeLa cells. The Tol2-based transposon donor vector (pT2A) and the Tol2 transposase expression vector (pCAGGS-T2TP) were kindly provided by Koichi Kawakami (National Institute of Genetics, Mishima, Japan) (Kawakami and Noda, 2004). The cDNAs of rtTA (Roney *et al.*, 2016) synthesized by FASMAC, tet-response element, and multiple cloning sites were inserted into a Tol2 donor vector by ligation and Gibson assembly to generate pTol2puro-rtTA2-TRE-MCS. The cDNAs of monomeric enhanced green fluorescent protein (mEGFP), CAHS1-mEGFP, CAHS3-mEGFP, CAHS8-mEGFP, and CAHS12-mEGFP were subcloned into the pT2Apuro-rtTA2-TRE-MCS vector.

### Cell culture

HeLa cells were kindly provided by Michiyuki Matsuda (Kyoto University). HeLa cells were maintained in Dulbecco’s Modified Eagle’s Medium (DMEM) high glucose (#08458-45, Nacalai Tesque, Kyoto, Japan) supplemented with 10% heat-inactivated fetal bovine serum (FBS) (#F7524, Sigma-Aldrich, St. Louis, MO, USA) at 37°C in a humidified atmosphere containing 5% CO_2_.

### Establishment of stable cell lines

For transposon-mediated gene transfer, HeLa cells were transfected with Tol2 donor vectors and Tol2 transposase-expressing vectors in a 1:1 ratio. Approximately 16–24 h after transfection, the cells were treated with 1.0 μg/mL puromycin (#ant-pr-1, InvivoGen, San Diego, CA, USA) for more than one week to select bulk cell lines. Cells were maintained in the presence of puromycin. In Fig. 2, the bright fluorescent cells were sorted into a 96-well plate using a cell sorter (MA900, SONY, Tokyo, Japan), and the single cell clone was used in Fig. 2. The bulk cell population was used in Fig. 3.

### Time-lapse imaging with hyperosmotic stress

HeLa cells were seeded on a 35-mm dish (#150460, Thermo Fisher Scientific, Rockford, IL, USA) or a 6 well plate (#140675, Thermo Fisher Scientific). The plasmid expressing the CAHS1-mEGFP protein, the CAHS3-mEGFP, the CAHS8-mEGFP, the CAHS12-mEGFP, the LEAM-mEGFP, the MAHS-mEGFP and the mEGFP were transfected into the HeLa cells with 293fectin^TM^ Transfection Reagent (#12347-019, Invitrogen, Waltham, MA, USA). After 24 h of transfection, the cells were loaded onto a microfluidic plate (M04S-03-5PK, Millipore, Burlington, MA, USA) according to the manufacturer’s instructions. The microfluidic plates were controlled by a CellASIC ONIX2 Microfluidic System (CAX2-S0000, Millipore). After 48 h of transfection, the culture medium was replaced with an imaging medium containing FluoroBrite^TM^ DMEM (#A1896701, Thermo Fisher Scientific) supplemented with 1% GlutaMAX^TM^ Supplement (#35050061, Thermo Fisher Scientific) and 0.1% bovine serum albumin. The imaging medium continuously flowed at 5 psi (“psi” is a unit of pressure) while heating at 37°C by a temperature-controllable manifold (CAX2-MXT20, Millipore).

For hyperosmotic shock experiments, 0.5 M sorbitol solution and 0.2 M sodium chloride solution were prepared as follows: 4.5 M sorbitol in DDW and 5 M sodium chloride in DDW were dissolved by the imaging medium, resulting in 0.5 M sorbitol solution and 0.2 M sodium chloride solution, respectively. The transfected cells seeded on microfluidic plates were time-lapse imaged under the imaging medium condition every 10 sec for 2 min. The imaging medium continuously flowed at 1 psi while heating at 37°C. Then, 0.5 M sorbitol solution or 0.2 M sodium chloride solution was added, and fluorescence images were acquired every 10 sec for 5 min. The hyperosmotic medium continuously flowed at 5 psi while heating at 37°C. After incubation, the hyperosmotic medium was replaced with the imaging medium, and the cells were observed every 10 sec for 10 min. The imaging medium continuously flowed at 5 psi while heating at 37°C.

Cells were imaged with an inverted microscope (IX83, Olympus/Evident, Tokyo, Japan) equipped with an sCMOS camera (ORCA-Fusion BT, Hamamatsu photonics, Hamamatsu, Japan), a spinning disk confocal unit (CSU-W1, Yokogawa Electric Corporation, Musashino, Japan), and diode lasers at a wavelength of 488 nm. An oil immersion objective lens (UPLXAPO100XO, N.A. 1.45, Olympus/Evident) was used. The excitation laser and fluorescence filter settings were as follows: excitation laser, 488 nm; dichroic mirror (DM), 405/488/561 nm; and emission filters, 500–550 nm. During the observation, the cells were incubated with microfluidic plates at 37°C. The microscope was controlled by MetaMorph software (Molecular Devices, San Jose, CA, USA).

Fluorescence imaging data were analyzed and quantified in Fiji/ImageJ (Schindelin *et al.*, 2012). The background was subtracted by a constant value. If salt-and-pepper noise was not negligible, a median filter with 1-pixel was applied. To compensate for the misregistration caused during time-lapse imaging, the “StackReg function” was applied with the “Translation” option.

### Fluorescence imaging for quantifying expression levels

HeLa cells carrying the doxycycline-inducible expression cassette of CAHS1, 3, 8, 12-mEGFP or mEGFP were seeded on a 96-well plate (#167008, Thermo Fisher Scientific). One day after seeding, the cells were treated with the indicated concentration of doxycycline, and 24 h after doxycycline treatment, the cells were observed with a fluorescence microscope.

The cells were imaged with an inverted microscope (IX81, Olympus/Evident) equipped with a cooled CCD camera (Retiga 4000R, Photometrics, Tucson, AZ, USA), an excitation light source (Spectra-X light engine, Lumencor Inc., Beaverton, OR, USA), a laser-based autofocus system (IX2-ZDC, Olympus/Evident), a controller for filter wheels and XY stage (MAC5000, Ludl Electronic Products, Hawthorne, NY, USA), and an incubation chamber (STXG-IX3WX, Tokai Hit, Fujinomiya, Japan). An objective lens (UPlanApo 10x, N.A. 0.40, Olympus/Evident) was used. For EGFP imaging, a 475/28 excitation filter, a 20/80 beamsplitter dichroic mirror (Chroma Technology Corp., Bellows Falls, VT, Japan), and a 542/27 emission filter (Semrock, IDEX Health & Science, LLC, West Henrietta, NY, USA) were used. During the observation, live cells were incubated in a stage incubator containing 5% CO_2_ at 37°C. The microscope was controlled by MetaMorph software (Molecular Devices).

The fluorescence imaging data were analyzed and quantified in Fiji/ImageJ (Schindelin *et al.*, 2012). The background was subtracted by the rolling ball method. The region of interest (ROI) of each cell was extracted with the “StarDistD2” function (Probability/Score Threshold: 0.55), and the fluorescence intensity (Mean value) of each ROI was obtained with the “Measure” function.

### Cell viability assay and cell death assay

For the cell viability assay and the cell death assay, a low serum concentration medium (1% FBS), hereinafter referred to as the assay medium, was used to reduce the background absorbance in the measurement of lactate dehydrogenase (LDH) release assay because serum contains LDH. The assay medium contains 1x DME culture solution (#639-29631, Nitta Gelatin, Yao, Japan), 1% FBS, 26 mM NaHCO_3_ (#09655-25, Nacalai Tesque), and 1 μg/mL puromycin (if needed). For hyperosmotic stress, a 3.2 M sorbitol solution in the assay medium was prepared by mixing 4.5 M sorbitol in DDW, 5x DME culture solution, 260 mM NaHCO_3_, 100% FBS, DDW, and 1 mg/mL puromycin. This 3.2 M sorbitol solution in the assay medium was serially diluted with the assay medium to produce the indicated concentration of sorbitol solution. For drug-inducible gene expression, 1 mg/mL doxycycline (#D4116, Tokyo Chemical Industry, Tokyo, Japan) was serially diluted with the assay medium to produce the indicated concentration of doxycycline solution in the assay medium.

HeLa cells were plated on 96-well plates (7.5 × 10^2^ – 3.0 × 10^3^ cells/well) (#167425, Thermo Fisher Scientific), and cultured in the assay medium for 24 h. To induce gene expression, the cells were treated with doxycycline for 24 h, followed by the treatment with sorbitol solution for 24 h. For the simultaneous cell viability assay and cell death assay (Fig. 2), half of the cell supernatant was used for the water-soluble tetrazolium (WST)-1 assay with a Premix WST-1 Cell Proliferation Assay System (#MK400, TaKaRa, Otsu, Japan) and the other half for the lactate dehydrogenase (LDH) release assay with an LDH Cytotoxicity Detection Kit (#MK401, TaKaRa) according to the manufacturer’s instructions. Both the WST-1 and LDH release assays were evaluated based on absorbance at 450 nm (and 620 nm as a control) using a microplate reader (#51119000, Thermo Fisher Scientific).

The absorbance values obtained from the microplate reader were exported and analyzed by Python (ver. 3.7.3). First, the absorbance values were subtracted from the value obtained from the well containing only the assay medium. Second, the subtracted values were normalized by the following equation:


Y = (X-Low)/(High-Low)

where *Y* is the normalized value, *X* is the experimental data, and *High* and *Low* are the highest and lowest values of the data obtained on the same day, respectively. For the cell viability assay in Fig. 2, the High and Low values were obtained from the maximum and minimum values on the same day, respectively. In Fig. 3, where the bulk population cell lines were used, the High and Low values were obtained from the value of the well in the absence of sorbitol and the minimum values on the same day, respectively. For the cell death assay, the High and Low values were taken from the value using Triton X-100 treated cells and the lowest values on the same day, respectively. Of note, the absolute number of cells seeded on the well plate varied between experiments, even though the number of cells was counted and the same number of cells were seeded between experiments. Therefore, the “High” and “Low” values were changeable. In addition, the LDH assay requires the supernatant to be taken from each well. These factors and procedures would cause the large variation between experiments. The normalized values were used to generate maps using the seaborn library in Python (Figs. 2D, 3B, S1, S2 and S3). The normalized data were fitted with the following functions using the scipy.optimize.corve_fit in Python:


$$WST(x)=(max-min)*{IC50}^{n}/(x^{n}+IC50^{n})+min$$

$$LDH(x)=(max-min)*x^{n}/(x^{n}+EC50^{n})+min$$

where *WST(x)* and *LDH(x)* are the fitted WST and LDH values, *x* is the sorbitol concentration, max and min are the maximum and minimum values, *n* is the Hill coefficient, and *IC50* and *EC50* are the half inhibitory concentration and half effective concentration for cell viability and cell death, respectively.

### Structure prediction by AlphaFold2

The predicted structures of CAHS1, CAHS3, CAHS8, and CAHS12 were obtained from the AlphaFold Protein Structure Database (https://alphafold.ebi.ac.uk/). Direct links are as follows: CAHS1 (https://alphafold.ebi.ac.uk/entry/J7M799), CAHS3 (https://alphafold.ebi.ac.uk/entry/J7M3T1), CAHS8 (https://alphafold.ebi.ac.uk/entry/A0A1D1UQN2), and CAHS12 (https://alphafold.ebi.ac.uk/entry/A0A1D1VNN8).

## Results and Discussion

### Hyperosmotic stress-induced changes in the subcellular localization of CAHS, LEAM, and MAHS proteins in HeLa cells

We examined changes in the subcellular localization of anhydrobiosis-related proteins in *R. varieornatus* induced by hyperosmotic stress, which is thought to be an early step in anhydrobiosis. Six types of proteins, including CAHS1, CAHS3, CAHS8, and CAHS12, mitochondrial LEA (LEAM), and mitochondrial abundant heat soluble (MAHS), were separately expressed as mEGFP-fused protein in HeLa cells (Fig. 1A). We selected CAHS1, CAHS3, CAHS8, and CAHS12 because they reversibly form different types of aggregates in response to osmotic stimulation (Tanaka *et al.*, 2022; Yagi-Utsumi *et al.*, 2021), but their roles in osmotic stress resistance have not been investigated except for CAHS3 (Tanaka *et al.*, 2022). The cells were loaded into a microfluidic chamber for rapid osmolyte solution perfusion. One day after loading into the microfluidic chamber, the cells were imaged with a spinning disk conformal microscope to visualize the subcellular distribution of these fusion proteins upon hyperosmotic stress such as 0.5 M sorbitol or 0.2 M NaCl (Fig. 1B). Of note, NaCl is the principal determinant of tonicity of the extracellular fluid (Burg *et al.*, 2007). However, cell membranes are permeable to Na^+^, and elevated chloride specifically increases the expression of α-Na^+^-K^+^-ATPase (Capasso *et al.*, 2003). For this reason, poorly permeating organic solutes like sorbitol, mannitol, or raffinose are often used to produce hypertonicity (Burg *et al.*, 2007).

HeLa cells expressing mEGFP treated with the osmolytes showed increased fluorescence signal in both the cytoplasm and nucleus, and puncta formation in the nucleus (Fig. 1C and Movie 1). These changes were reverted to the basal state by osmolyte washout (Fig. 1C). Under unstressed conditions, CAHS1-mEGFP, CAHS8-mEGFP, and CAHS12-mEGFP were broadly distributed in both the cytosol and nucleus, whereas CAHS3-mEGFP was mainly localized in the cytosol (Fig. 1D–G and Movies 2–5). When exposed to hyperosmotic medium supplemented with 0.5 M sorbitol or 0.2 M NaCl, these CAHS proteins formed different aggregation patterns; CAHS1-mEGFP mainly forms granules and filamentous structures and slightly accumulates at the plasma membrane (Fig. 1D and Movie 2). CAHS3-mEGFP shows fibrous structures in the cytoplasm (Fig. 1E and Movie 3). CAHS8-mEGFP seems to have the same pattern as mEGFP, but it slightly accumulates at the nuclear membrane (Fig. 1F and Movie 4). CAHS12-mEGFP appears to undergo decomposition in the nucleus (Bracha *et al.*, 2018), while it exhibits granules and filamentous structures (Fig. 1G and Movie 5). These results are, for the most part, consistent with previous reports (Tanaka *et al.*, 2022; Yagi-Utsumi *et al.*, 2021). Interestingly, CAHS proteins appear to behave differently between 0.5 M sorbitol and 0.2 M NaCl osmotic stress. For example, CAHS1 did not aggregate at the cell membrane under 0.2 M NaCl stress, but showed strong aggregation in the cytoplasm (Fig. 1D). CAHS12 also showed strong aggregation at the nuclear membrane and plasma membrane upon 0.5 M sorbitol and 0.2 M NaCl, respectively (Fig. 1G). Regardless of whether the aggregate forms granules or filaments during hyperosmotic stress, mEGFP and all CAHS protein condensates show rapid disassembly and restoration of coherent distribution upon the release of stress. It is reasonable to conclude that these rapid aggregation and disassembly processes are attributed to the biophysical properties of both mEGFP and each CAHS protein. Such efficient reversible phase transitioning mirrors the relatively fast induction and recovery of tardigrade anhydrobiosis. The varying modes of aggregation and localization patterns of different CAHS paralogs upon hyperosmotic stress suggest that these paralogs serve differentiated functions rather than for mere redundancy. Emerging tissue-specific expression patterns of tardigrade anhydrobiosis-related proteins (Tanaka *et al.*, 2023) could be part of the reasons for such differentiation.

It has been reported that two mitochondrial heat-soluble proteins, LEAM and MAHS, in *R. varieornatus* improve the hyperosmotic tolerance of human cells, possibly by acting as mitochondrial protectants (Tanaka *et al.*, 2015). Therefore, we investigated the subcellular localization of LEAM and MAHS in HeLa cells stimulated by hyperosmotic stress. Under unstressed conditions, LEAM-mEGFP and MAHS-mEGFP were localized to the mitochondria, and LEAM-mEGFP was weakly distributed in the cytosol and nucleus (Fig. 1H and I). Hyperosmotic stress arrested the random movement of mitochondria, but did not substantially change the subcellular localization of LEAM-mEGFP and MAHS-mEGFP (Fig. 1H and I). Removal of osmolytes by replacement with an isosmotic medium resumed the thermal fluctuation of the mitochondria. Based on the fact that both LEAM and MAHS proteins localize to mitochondria and are highly hydrophilic (Tanaka *et al.*, 2015), LEAM and MAHS are likely to be localized to the mitochondrial matrix. Taken together, these results suggest that the resistance to hyperosmotic stress by LEAM and MAHS expression is not cell-wide protection as observed for CAHS proteins, so we focused on the CAHS proteins for our subsequent analyses.

### CAHS1 expression renders HeLa cells resistant to hyperosmotic stress

Recent studies have elucidated that the CAHS3 protein forms fibrillar aggregates under hyperosmotic conditions in mammalian cells, thereby enhancing their tolerance to hyperosmotic stress, as observed in *Drosophila* S2 cells (Tanaka *et al.*, 2022). However, the question remained as to whether other CAHS proteins, which exhibit varied aggregation patterns under elevated osmotic pressure, also contribute to augmented resistance to such conditions. To this end, we developed a method for quantitatively assessing osmotic stress resistance using HeLa cells expressing CAHS1 (Fig. 2A). We first established a single-cell clone of HeLa cells, expressing CAHS1-mEGFP in a manner dependent on doxycycline treatment. The experimental procedure was as follows: One day after cell seeding, doxycycline was added to induce CAHS1-mEGFP expression for two days, followed by different concentrations of sorbitol as osmolite. Cell viability and increased cell death were evaluated using the WST-1 and LDH assays, respectively (see Methods for details)(Fig. 2B).

We first confirmed the increase in CAHS1-mEGFP expression levels when we treated the cells with doxycycline (Fig. 2C). Despite performing single-cell cloning, the variation in the expression levels of CAHS1-mEGFP between cells was unexpectedly large. Under this condition, the cells were further exposed to hyperosmotic stress. Cell viability was strongly inhibited even in 0.1 M sorbitol in the absence of doxycycline (Fig. 2D, left, first row, and Fig. S1). The increase in CAHS1-mEGFP expression attenuated the decrease in cell viability by hyperosmotic stress with sorbitol (Fig. 2D, left, and Fig. S1). The half maximal inhibitory concentration (IC50) increased depending on the CAHS1-mEGFP expression level (Fig. 2E, left). Parental HeLa cells as a negative control showed no change in cell viability with or without doxycycline treatment (Fig. 2E, right). In line with the cell viability assay, the number of hyperosmotic stress-induced cell death was also reduced by the expression of CAHS1-mEGFP (Fig. 2D, right, and Fig. S1). The half-maximal effective concentration (EC50) demonstrated a tendency to elevate with increasing expression of CAHS1-mEGFP (Fig. 2F, left). As a negative control, doxycycline treatment did not affect the EC50 values of sorbitol-induced cell death in parental HeLa cells (Fig. 2F, right). These results suggest that CAHS1 expression confers resistance to hyperosmotic stress in HeLa cells. CAHS1-mEGFP localizes to aggregates and fibrous structures and even to the plasma membrane at high osmotic pressure (Fig. 1D), suggesting that CAHS1 exerts some protective function against osmotic stress at these sites. It should be noted that the time-scales of CAHS1 aggregation (~5 min) is much faster than the inhibition of cell proliferation and cell death (~24 h) in response to hyperosmotic stress. This difference may explain the non-linear relationship between CAHS1 expression level (Fig. 2C) and the effects of sorbitol on cell viability (Fig. 2E) and cell death (Fig. 2F). In addition, in this study, the single-cell clone of HeLa cells stably expressing CAHS1-mEGFP indicated lower IC50 values for cell viability and EC50 values for cell death in response to sorbitol stimulation in the absence of doxycycline than parental HeLa cells (Fig. 2E and 2F). This may be because the single-cell clones established in this study happen to retain such properties. Further investigation would be needed in future studies to address this concern.

### Expression of CAHS1, CAHS3, or CAHS8 confers osmotic stress resistance to HeLa cells

We next examined the other CAHS proteins for their ability to confer osmotic stress tolerance to HeLa cells. To simplify the experiment, HeLa cell lines were established that express mEGFP, CAHS1-mEGFP, CAHS3-mEGFP, CAHS8-mEGFP, and CAHS12-mEGFP in a doxycycline-dependent manner. Of note, these HeLa cell lines were bulk population cells, but not single-cell clones, because it takes longer time to establish the single-cell clones. First, we quantified the CAHS expression levels induced by doxycycline treatment in these HeLa cells, and found that doxycycline treatment elevated CAHS expression levels several-fold (Fig. 3A). The greater cellular heterogeneity compared to the single-cell cloned CAHS1-mEGFP cell line (Fig. 2C) may be because these cell lines are bulk population cells that have not been subjected to single-cell cloning. Second, we examined the changes in cell viability in these cell lines upon hyperosmotic stress with sorbitol using the WST-1 assay. The average cell viability of each cell line under conditions without and with doxycycline is shown in the heat map (Fig. 3B). Even before doxycycline treatment, cell viability was higher in cells expressing CAHS1-mEGFP and CAHS8-mEGFP, but not CAHS3-mEGFP and CAHS12-mEGFP, than in control mEGFP-expressing cells (Fig. 3B, left). This may be because leakage of CAHS1 or CAHS8 proteins prior to doxycycline treatment contributes to tolerance to high osmotic pressure, while the expression levels of CAHS3 and CAHS12 did not reach the critical thresholds. In addition, doxycycline treatment rendered CAHS1-mEGFP, CAHS3-mEGFP, or CAHS8-mEGFP expressing cells more resistant to higher concentrations of sorbitol than control mEGFP-expressing cells (Fig. 3B, right). In our hand, CAHS1-mEGFP, CAHS3-mEGFP, CAHS8-mEGFP, or CAHS12-mEGFP expressing cells have rather increased cell viability under low concentrations of sorbitol (Fig. 3B, right). At this time, we do not know whether this is a result of variability in our experiments or whether the CAHS proteins potentiate cell viability under low concentrations of sorbitol. To quantitatively evaluate the resistance to hyperosmotic stress, we calculated the ratio of the IC50 value of cell viability against sorbitol after doxycycline treatment to that before doxycycline treatment. The IC50 values of CAHS1-mEGFP, CAHS3-mEGFP, and CAHS8-mEGFP cells increased by 10–20% in a doxycycline-dependent manner (Fig. 3C). No increase in the ratio of IC50 values was observed in mEGFP or CAHS12-mEGFP cells. Although the *p*-value (*p* = 0.058 for CAHS1 compared with mEGFP) indicated that the observed difference was not statistically significant, the data suggest a potential trend toward increased resistance to osmotic stress through CAHS1 expression, which may warrant further investigation. The expression level of CAHS12 remained relatively low compared to CAHS3 and CAHS8, which had comparable expression levels before doxycycline treatment (Fig. 3A). We have previously shown that CAHS1 condensation displays concentration-dependent sigmoidal threshold-like behavior between 0.3–0.6 mM at neutral pH (Tanaka *et al.*, 2022; Yagi-Utsumi *et al.*, 2021); therefore, there still remains the possibility that the CAHS12 concentration did not exceed the critical threshold. Nevertheless, from these results, we conclude that overexpression of CAHS1, CAHS3, and CAHS8 moderately confers tolerance to hyperosmotic stress in HeLa cells, and it is regardless of the mode of aggregation, whether granular or fibrous.

**Fig. 4:**
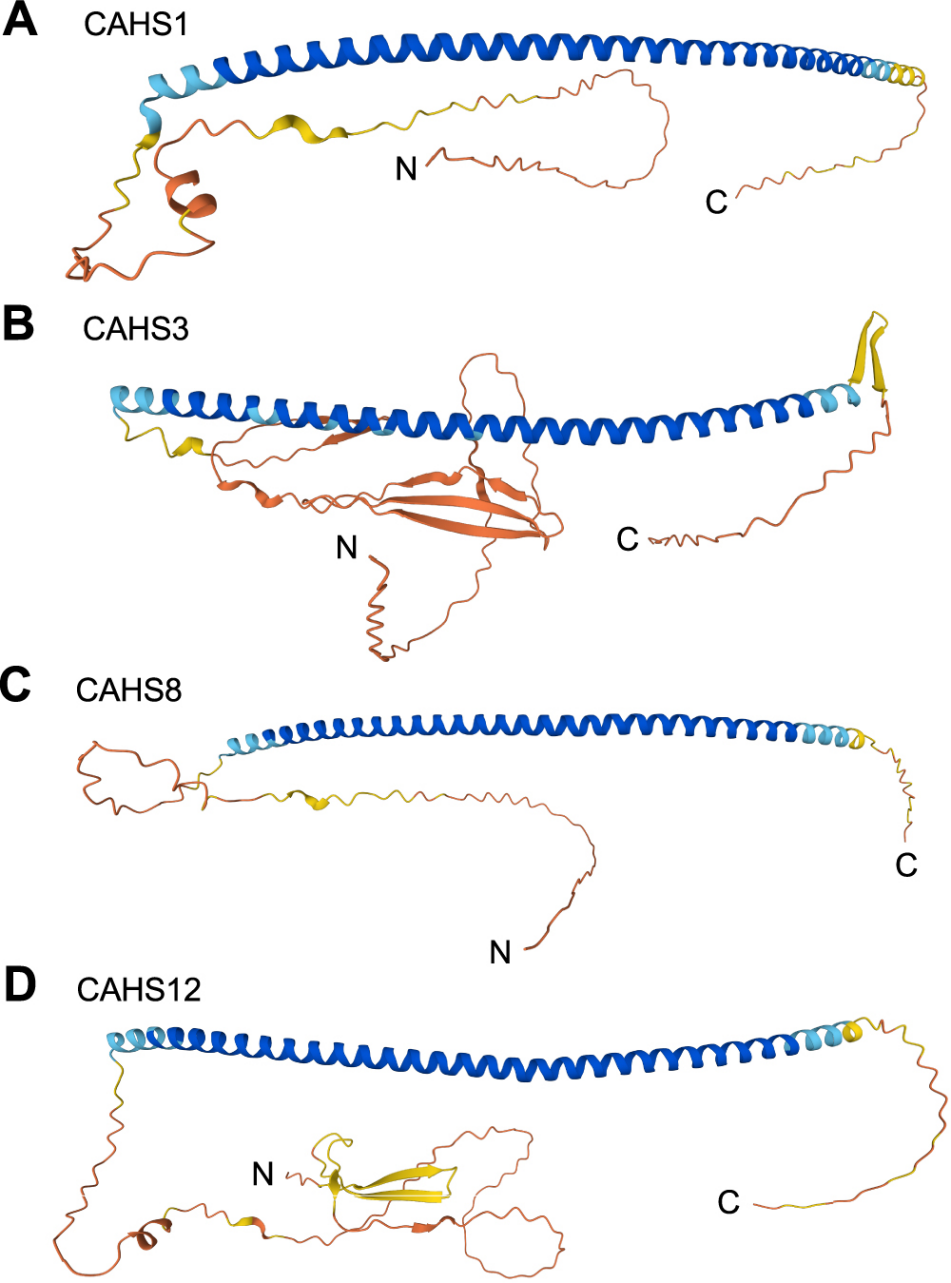
The predicted structure of CAHS1, CAHS3, CAHS8, and CAHS12 The predicted structure of CAHS1 (A), CAHS3 (B), CAHS8 (C), and CAHS12 (D) by AlphaFold2 are shown. A per-residue model confidence score (pLDDT) produced by AlphaFold are represented as colors; blue, pLDDT>90; cyan, 90>pLDDT>70; yellow, 70>pLDDT 50; orange, pLDDT<50.

Finally, we investigated the structure of the CAHS proteins in AlphaFold (Jumper *et al.*, 2021; Varadi *et al.*, 2022), and found that CAHS1, CAHS3, CAHS8, and CAHS12 all share a single long α-helix and intrinsically disordered regions (IDRs) at the N- and C-termini (Fig. 4); CAHS3 and CAHS12 have a predicted structure at the N-terminus, but their confidence is low. Despite the close structural similarity between CAHS proteins, the subcellular localization of these CAHS proteins under high osmotic pressure differed from each other (Fig. 1). As shown in the previous report, CAHS3 shows a coiled-coil structure and forms a fibrous structure, thereby stabilizing the cell structure and increasing the resistance to high osmotic pressure (Tanaka *et al.*, 2022). We have demonstrated that CAHS1 and CAHS8 localize to granules, plasma membrane, and nuclear membrane at high osmotic pressure (Fig. 1), suggesting that CAHS1 and CAHS8 may protect organelles such as plasma membrane and nuclear membrane from hyperosmotic stimuli. These diverse aggregation patterns of CAHS proteins may coordinate anhydrobiosis in tardigrades.

## Conclusion

In this study, we expressed *R. varieornatus* CAHS proteins in HeLa cells to investigate their effects on resistance to hyperosmotic stress, an early step in anhydrobiosis. All of these proteins demonstrated reversible aggregation upon hyperosmotic stress in HeLa cells, exhibiting granular and filamentous structures (Fig. 1). Furthermore, we found that expression of CAHS1, CAHS3, and CAHS8 conferred resistance to sorbitol-induced hyperosmotic stress in HeLa cells (Figs. 2 and 3). These results not only provide new insights into the mechanism of desiccation tolerance in *R. varieornatus*, but also suggest a potential use of these CAHS proteins as cryoprotectants.

## Fundings

This work was financially supported in part by grants from the MEXT/JSPS KAKENHI (22H02625 and 24H01416) to K. Aoki and to K. Arakawa (21H05279), the Takeda Science Foundation to K. Aoki, the NAGASE Science Technology Foundation to K. Aoki, and the Joint Research of the Exploratory Research Center on Life and Living Systems (ExCELLS) (ExCELLS program No. 19-501, 24EXC601) to K. Arakawa and K. Aoki, and partly by research funds from the Yamagata Prefectural Government and Tsuruoka City, Japan to K. Arakawa.

## Data Availability

The supporting information for this article is available in J-STAGE Data.

## Abbreviations

CAHS: cytoplasmic abundant heat soluble
LEA: late embryogenesis abundant
LEAM: late embryogenesis abundant protein mitochondrial
MAHS: mitochondrial abundant heat soluble
mEGFP: monomeric enhanced green fluorescent protein
psi: pound per square inch
WST: water-soluble tetrazolium
LDH: lactate dehydrogenase
Dox: doxycycline
IC50: half inhibitory concentration
EC50: half effective concentration
IDR: intrinsically disordered region

## Supplementary Material

Movie 1. HeLa cells expressing mEGFP were loaded onto a microfluidic plate and grown for 1 day. Time-lapse imaging was performed using a spinning disk confocal microscope. The cells were treated with 0.5 M Sorbitol (left) and 0.2 M NaCl (right) at 2 min and incubated for 5 min, followed by the replacement of imaging medium. Images were acquired every 10 sec. Total imaging time = 17 min.

Movie 2. HeLa cells expressing CAHS1-mEGFP were loaded onto a microfluidic plate and grown for 1 day. Time-lapse imaging was performed using a spinning disk confocal microscope. The cells were treated with 0.5 M Sorbitol (left) and 0.2 M NaCl (right) at 2 min and incubated for 5 min, followed by the replacement of imaging medium. Images were acquired every 10 sec. Total imaging time = 17 min.

Movie 3. HeLa cells expressing CAHS3-mEGFP were loaded onto a microfluidic plate and grown for 1 day. Time-lapse imaging was performed using a spinning disk confocal microscope. The cells were treated with 0.5 M Sorbitol (left) and 0.2 M NaCl (right) at 2 min and incubated for 5 min, followed by the replacement of imaging medium. Images were acquired every 10 sec. Total imaging time = 17 min.

Movie 4. HeLa cells expressing CAHS8-mEGFP were loaded onto a microfluidic plate and grown for 1 day. Time-lapse imaging was performed using a spinning disk confocal microscope. The cells were treated with 0.5 M Sorbitol (left) and 0.2 M NaCl (right) at 2 min and incubated for 5 min, followed by the replacement of imaging medium. Images were acquired every 10 sec. Total imaging time = 17 min.

Movie 5. HeLa cells expressing CAHS12-mEGFP were loaded onto a microfluidic plate and grown for 1 day. Time-lapse imaging was performed using a spinning disk confocal microscope. The cells were treated with 0.5 M Sorbitol (left) and 0.2 M NaCl (right) at 2 min and incubated for 5 min, followed by the replacement of imaging medium. Images were acquired every 10 sec. Total imaging time = 17 min.
