# Supplementary material for: Possible roles of CAHS proteins from Tardigrade in osmotic stress tolerance in mammalian cells
Source: Cell Struct Funct. 2024 Nov 19;49(2):123–33. doi: 10.1247/csf.24035 (PMC11930778; doi:10.1247/csf.24035)
Supplement: Supplementary file 6 — Supplementary Materials [file csf_49_24035_6.pdf]

Supplementary Materials

Supplementary Figure S1

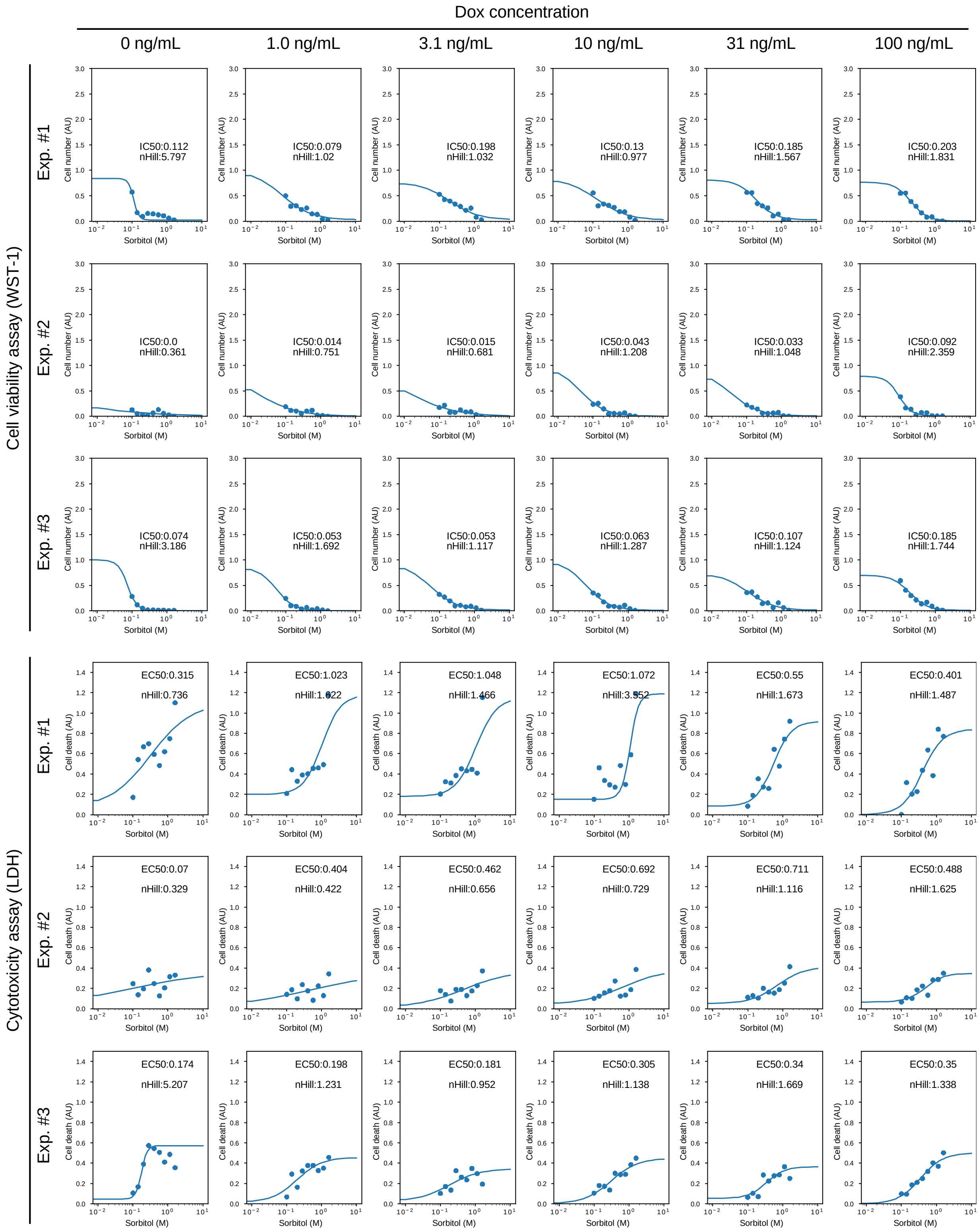

Figure S1. Cell viability and cytotoxicity in HeLa cells expressing CAHS1-mEGFP. The all data in Figure 2D (left) are shown with the fitted parameters.

Supplementary Figure S2

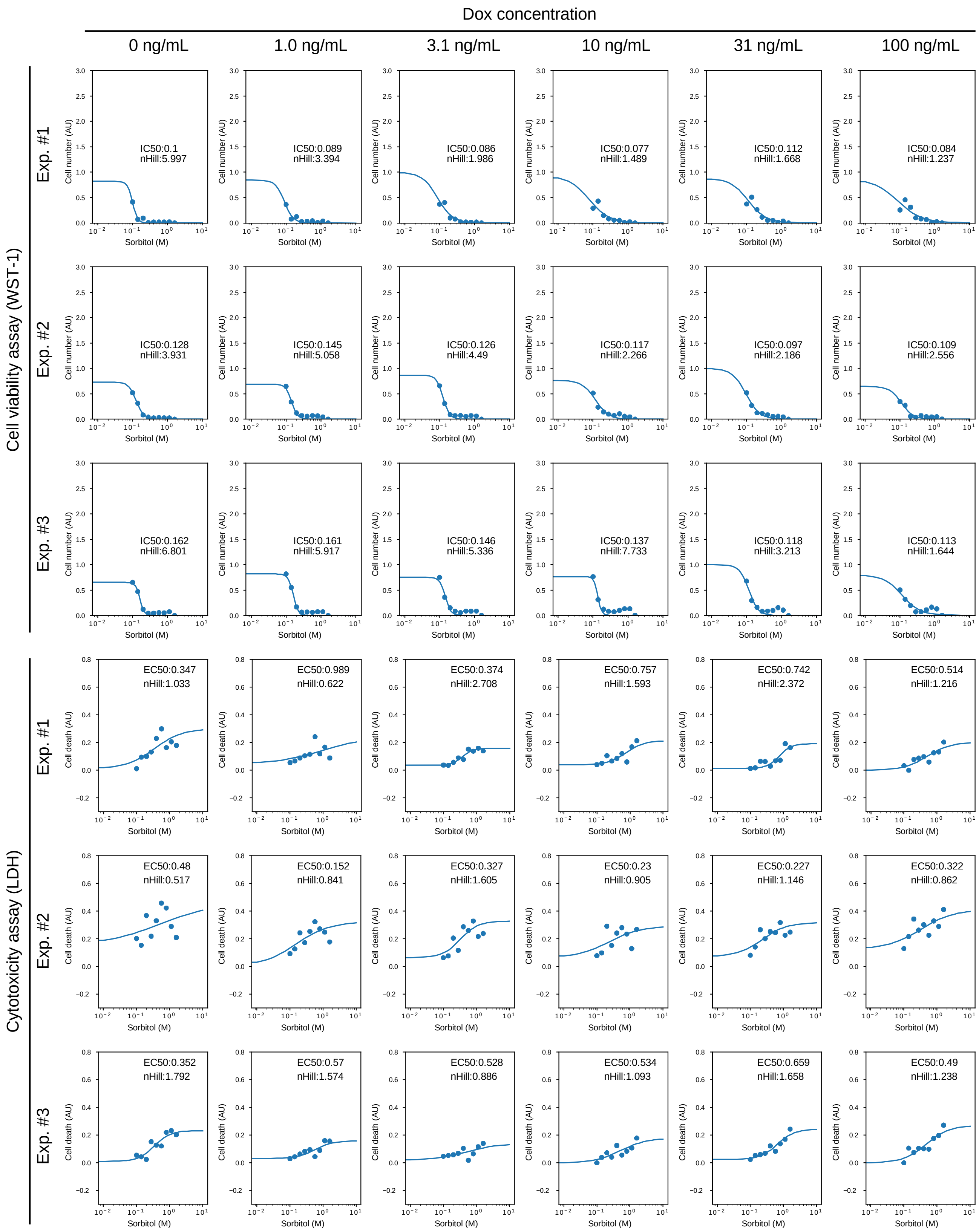

Figure S2. Cell viability and cytotoxicity in parental HeLa cells. The all data in Figure 2D (right) are shown with the fitted parameters.

Supplementary Figure S3

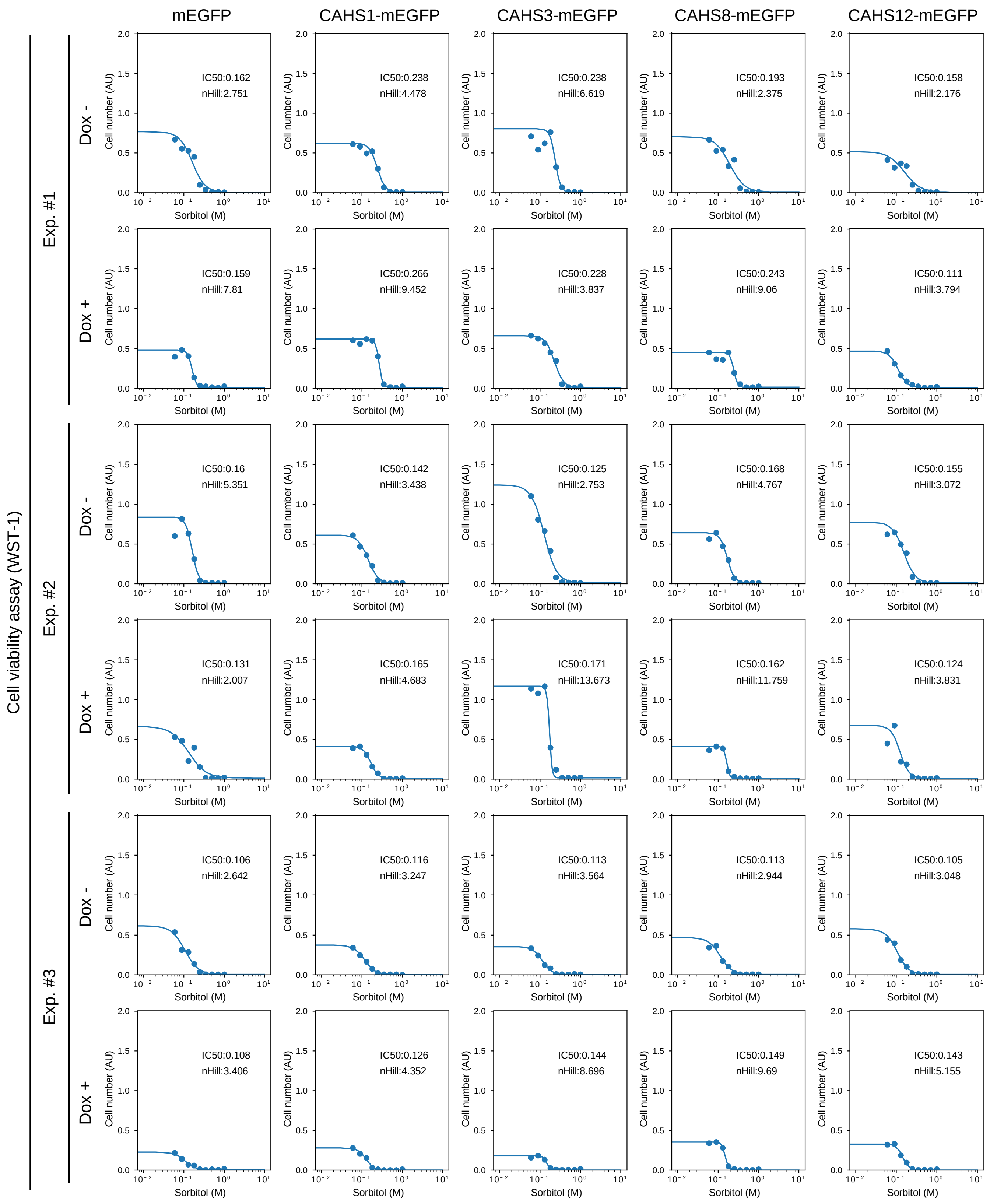

Figure S3. Cell viability in HeLa cells expressing mEGFP, CAHS1-, CAHS3-, CAHS8-, or CAHS12-mEGFP. The all data in Figure 3B are shown with the fitted parameters.

Table S1

Plasmid name	Figure	Source	Benchling Link
pCAGGS-mEGFP	1C	This study	https://benchling.com/s/seq-2DoIxGCleqxXSRrXPpCG
pCAGGS-CAHS1-mEGFP	1D	This study	https://benchling.com/s/seq-fNpWUGViSMU0EUO7pJKR
pCAGGS-CAHS3-mEGFP	1E	This study	https://benchling.com/s/seq-yQAT7ohPffRaNFpPGQ9p
pCAGGS-CAHS8-mEGFP	1F	This study	https://benchling.com/s/seq-B1eBVMip1UlguGoDi82M
pCAGGS-CAHS12-mEGFP	1G	This study	https://benchling.com/s/seq-IOf73DdxTcZxyEzEDNFJ
pCAGGS-LEAM-mEGFP	1H	This study	https://benchling.com/s/seq-57TG1q7c5ad8TshWcFWQ
pCAGGS-MAHS-mEGFP	1I	This study	https://benchling.com/s/seq-zOvLLNM8RG6Si8n3nA2F
pT2Apuro-rtTA2-TRE-mEGFP	3A, 3B, 3C, S3	This study	https://benchling.com/s/seq-eJBP2md59OAFr8qlMTR5
pT2Apuro-rtTA2-TRE-CAHS1-mEGFP	2C, 2D, 2E, 2F, 3A, 3B, 3C, S1, S2, S3	This study	https://benchling.com/s/seq-i1Oqs1PV4CIACqNTHZQf
pT2Apuro-rtTA2-TRE-CAHS3-mEGFP	3A, 3B, 3C, S3	This study	https://benchling.com/s/seq-pqVeh3bCZF3cXGGgCCI7
pT2Apuro-rtTA2-TRE-CAHS8-mEGFP	3A, 3B, 3C, S3	This study	https://benchling.com/s/seq-PBVZ1TtMD1quil3xlkTc
pT2Apuro-rtTA2-TRE-CAHS12-mEGFP	3A, 3B, 3C, S3	This study	https://benchling.com/s/seq-NrTjl5LMnuPYPaI7682w

Table S1. Plasmid list used in this study.

Movie 1

HeLa cells expressing mEGFP were loaded onto a microfluidic plate and grown for 1 day. Time-lapse imaging was performed using a spinning disk confocal microscope. The cells were treated with 0.5 M Sorbitol (left) and 0.2 M NaCl (right) at 2 min and incubated for 5 min, followed by the replacement of imaging medium. Images were acquired every 10 sec. Total imaging time = 17 min.

Movie 2

HeLa cells expressing CAHS1-mEGFP were loaded onto a microfluidic plate and grown for 1 day. Time-lapse imaging was performed using a spinning disk confocal microscope. The cells were treated with 0.5 M Sorbitol (left) and 0.2 M NaCl (right) at 2 min and incubated for 5 min, followed by the replacement of imaging medium. Images were acquired every 10 sec. Total imaging time = 17 min.

Movie 3

HeLa cells expressing CAHS3-mEGFP were loaded onto a microfluidic plate and grown for 1 day. Time-lapse imaging was performed using a spinning disk confocal microscope. The cells were treated with 0.5 M Sorbitol (left) and 0.2 M NaCl (right) at 2 min and incubated for 5 min, followed by the replacement of imaging medium. Images were acquired every 10 sec. Total imaging time = 17 min.

Movie 4

HeLa cells expressing CAHS8-mEGFP were loaded onto a microfluidic plate and grown for 1 day. Time-lapse imaging was performed using a spinning disk confocal microscope. The cells were treated with 0.5 M Sorbitol (left) and 0.2 M NaCl (right) at 2 min and incubated for 5 min, followed by the replacement of imaging medium. Images were acquired every 10 sec. Total imaging time = 17 min.

Movie 5

HeLa cells expressing CAHS12-mEGFP were loaded onto a microfluidic plate and grown for 1 day. Time-lapse imaging was performed using a spinning disk confocal microscope. The cells were treated with 0.5 M Sorbitol (left) and 0.2 M NaCl (right) at 2 min and incubated for 5 min, followed by the replacement of imaging medium. Images were acquired every 10 sec. Total imaging time = 17 min.
